# Mycoremediation and toxicity assessment of textile effluent pertaining to its possible correlation with COD

**DOI:** 10.1038/s41598-021-94666-8

**Published:** 2021-08-05

**Authors:** Geetanjali Rajhans, Adyasa Barik, Sudip Kumar Sen, Amrita Masanta, Naresh Kumar Sahoo, Sangeeta Raut

**Affiliations:** 1 Center for Biotechnology, School of Pharmaceutical Sciences, Siksha ‘O’ Anusandhan (Deemed to be University), Bhubaneswar, Odisha 751003 India; 2 Biostadt India Limited, Waluj, Aurangabad, Maharashtra 431136 India; 3 Department of Chemistry, ITER, Siksha ‘O’ Anusandhan (Deemed to be University), Bhubaneswar, Odisha 751003 India

**Keywords:** Biological techniques, Biotechnology, Microbiology, Environmental sciences

## Abstract

Globally, textile industries are one of the major sectors releasing dye pollutants. This is the first report on the positive correlation between toxicity and chemical oxygen demand (COD) of textile effluent along with the proposed pathway for enzymatic degradation of acid orange 10 using *Geotrichum candidum* within a very short stretch of time (18 h). Removal efficiency of this mycoremedial approach after 18 h in terms of chemical oxygen demand, biological oxygen demand, total suspended solids, salinity, color and dye concentration in the treated effluent reached to 98.5%, 56.3%,73.2%, 64%, 89% and 87% respectively. Also there was a decrease in pH of the treated effluent. FTIR analysis of the treated effluent confirmed biodegradation. The LCMS analysis showed the degradation of acid orange 10, which was confirmed by the formation of two biodegradation products, 7-oxo-8-iminonapthalene-1,3-disulfonate and nitrosobenzene, which subsequently undergoes stepwise hydrogenation and dehydration to form aniline via phenyl hydroxyl amine as intermediate. The X-ray diffraction studies showed that heavy metal content in the treated effluent has reduced along with decrease in % crystallinity, indicating biodegradation. The connection between toxicity and COD was also inveterated using Pearson’s correlation coefficient. Further the toxicological studies indicated the toxicity of raw textile effluent and relatively lower toxic nature of metabolites generated after biodegradation by *G. candidum*.

## Introduction

Urbanization and industrialization have paved the path for development of the many industries, including the textile industries. Clothing and textiles, after agriculture, is the basic requirement of human being. While the textile industry contributes worldwide economically, the environmental effects are due to high volumes of water use and the diversity and quantities of chemicals that are used in all manufacturing phases of textiles. The untreated effluent when disposed in the water bodies seriously impacted the people in the area^1^. Rivers and drainage bodies get loaded with precarious textile effluents that impact on the water quality, aquatic organisms and human life^2–7^. The diverse sort of dyes and chemicals used in textile manufacturing makes textile effluents very complex in terms of chemical compositions. According to previous records, in addition to dyes and its auxiliaries over 8000 chemicals are added such as several acids, salts, surfactants, metals, oxidizing and reducing agents^8^. These recalcitrants in untreated effluents are both harmful to marine and terrestrial organisms and have prolonged effects on health^9^.

To assess the performance of wastewater treatment facilities, the influent and treated water samples after each treatment phase (physical, chemical and biological) should be tracked. In general, microbial degradation or bioremediation is known to be a safe, natural, inexpensive and effective pollutant removal technique in the world^7,10–12^. Bioremediation is an innovative clean-up technology that involves the use of bacteria, fungi, actinomycetes, and earthworms^13^. Because of its low cost, high efficiency, and eco-friendly nature, it is a sustainable process for the treatment of organics-rich solid wastes and wastewater produced from various sources^14^. Bioremediation appears to be a promising alternative to other widely used clean-up technologies, such as photocatalytic degradation, because studies have shown that the intermediates released during the photocatalytic degradation process are detrimental to a variety of organisms in the environment^15–18^. Moreover, these processes are also associated with high-energy consumption and capital cost.

However, bacteria typically contribute to the breakdown of textile dyes creating and accumulating more intractable or hazardous aromatic amine substances that restrict their comprehensive applications to azo dye wastewater treatment plants^19^. Enhanced techniques are evidently the pre-condition for accelerated elimination of azo dyes, because any residual contaminants should be removed completely. Fungi have been investigated, especially those secreting non-specific oxidases which eventually lead to the azo dyes mineralization into CO_2_^20^. Fungal degradation (Mycoremediation) also leads to complete discoloration and detoxification, which prevents sludge removal and secondary contamination issues.

*Geotrichum* sp. is one of the few fungi found to degrade large amounts of artificial colors and molasses^21–26^. Because *Geotrichum* sp has received little attention, it is being used in the current study for the biodegradation and detoxification of textile effluent. In order to confirm the degradation efficiency of the fungus, toxicity evaluation is a requisite. The studies based on toxicity assessments could be done with bioassays using all forms of harmful compounds found in textile effluent and may well be utilized to evaluate the effect of unidentified compounds which cause detrimental, additive and synergistic effects^27,28^.

In the current study, an attempt to validate the non-toxicity of mycoremediated textile effluent as well as its biodegradation analysis such as FTIR, LCMS and XRD have been carried out. Furthermore, bioassays such as genotoxicity, phytotoxicity and microbial toxicity assays have also been conducted so that the toxicity level of raw and treated textile effluent could be assessed.

## Materials and methods

### Collection of samples

Samples of textile dyeing effluent have been obtained from a nearby textile factory in Khurda, India. During regular operations, factory workers sampled 100 mL of wastewater every two hours to ensure that the study involves variability in substances.

### Microbial culture conditions

This work utilizes *Geotrichum candidum,* a ubiquitous fungus belonging to *Dipodoascaceae* family. It was grown at 35 °C on Potato Dextrose Agar plates (PDA) (pH-5.6 ± 0.2). Pure fungal culture was inoculated in 3% malt extract broth after 24 h to maintain the strain and cultured at 35 °C, 100 rpm. Using DP media (dextrose and peptone in ratio of 2:3) at 35 °C and 100 rpm, the optimum fungal growth was achieved^25^.

### Analysis of conventional indicators of textile effluent

In this part of the analysis, the *G. candidum* culture was used to biodegrade the textile effluent. A conical flask containing raw textile effluent (25 ml) was inoculated with fungal culture (5%, v/v), followed by incubation at 35 °C, 100 rpm^25^. At regular intervals, aliquots were obtained from the flask and then centrifuged (10,000 × g) for 10 min. Thereafter, the conventional indicators for raw and treated effluent such as Chemical Oxygen Demand (COD), Biological Oxygen Demand (BOD), Total Suspended Solids (TSS), pH, salinity, color and concentration were assessed following the Standard Methods^29^. COD concentrations were measured using the potassium dichromate method; Pt–Co color scale was used to measure color. TSS estimation was carried out by simple laboratory method^30^. The pH of water was determined by using a glass electrode pH meter (Systronics India, Model 362) and salinity was measured by Hanna Salinity Tester (HI98319). Dye concentration of the sample was determined using colorimeter (Systonic, S-912). Every experiment has been carried out in triplicates and standard deviation has been presented with the average data.

The following equation (Eq. (1)) was used to quantify degradation as a percentage reduction of COD:

1 $${\mathrm{ \%COD}}_{\mathrm{reduction}}=\frac{{\mathrm{COD}}_{\mathrm{initial}}-{\mathrm{COD}}_{\mathrm{t}}}{{\mathrm{COD}}_{\mathrm{initial}}}\mathrm{ x }100$$

COD _initial_: initial value of COD. COD _t_: value of COD at time ‘t’ (h).

### Growth kinetics

The following Eq. (2) has been used to determine the specific growth rate of *G. candidum*.

2 $$ln\frac{x}{{x}_{o}}=\mu t$$

x: biomass concentration (g L^−1^) at ‘t’ time. x_o_: initial biomass concentration (g L^−1^) at ‘t_0_^’^ time. μ: specific growth rate (h^–1^).

The expression for growth yield (Y) is

3 $$\frac{dx}{ds}=Y$$

The Eq. 3 can be rewritten as follows ^31^:

4 $$x-{x}_{o}=Y({S}_{o}- S)$$

S_o_: initial substrate (COD) concentration (mg L^−1^). S: final substrate (COD) concentration (mg L^−1^). x: biomass concentration (mg L^–1^). x_o_: initial biomass concentration (mg L^−1^).

### Analytical studies

The metabolites formed in textile effluent after decolorization and degradation were obtained by same volume with ethyl acetate. The extract was dried over anhydrous sodium sulfate and evaporated to dryness in a rotary evaporator. The resulting crystals were dissolved in small volumes of methanol (HPLC grade), and then used for analysis such as FTIR (Fourier-transform infrared spectroscopy), LCMS (Liquid chromatography–mass spectrometry) and XRD (X-ray Diffraction).

The FTIR analysis of the effluent was performed with Attenuated total reflectance- Fourier transform infrared spectroscopy (ATR-FTIR, FT/IR-4600, JASCO, Japan). A drop from each sample was placed on a Zinc selenide (ZnSe) frame, and the spectra were documented with an average of 32 scans between the 4000 and 600 cm^−1^ spectral ranges.

The raw and treated samples were also analyzed using LCMS (Waters Micromass Q-Tof Micro) and the flow rate and temperature were maintained at 0.2 ml min^−1^ and 35 °C, respectively. The running time was 41 min. Two different solvents with varying proportions, such as water with 0.1% formic acid and acetonitrile with 0.1% formic acid were used. The deuterium lamp (DL) temperature was set at 250 °C with m/z value 50–1000 runs in the positive ion mode.

X-ray diffraction patterns before and after biodegradation of textile effluent were recorded using RIGAKU-ULTIMA IV, Japan diffractometer with monochromatic CuKα radiation (λ = 1.5406) over the range of 10–90° (2θ). The metals were identified with powder diffraction standard file (JCPDS, Joint Committee on Powder Diffraction Standards Newtown Square, Pennsylvania, USA).

### Toxicity study

#### Phytotoxicity

The phytotoxicity analysis was performed using *Phaseolus mungo* (at room temperature) on both raw and treated effluent. Simultaneously, the control set was conducted using tap water. After 7 days, toxicity of the raw and treated effluent was evaluated by the length of radical, plumule and germination percentage. Mean with standard deviation for all results were presented.

#### Microbial toxicity

A short-term toxicity test of textile effluent before and after treatment was demonstrated by exposing the bacteria *Escherichia coli* (ATCC 443) to the textile effluent for 15 min. Toxicity to *E. coli* was determined spectrophotometrically by evaluating the difference in the number of cells before and after treatment. All the tests were conducted in triplicate. An experiment with a control set was also carried out.

#### Genotoxicity Study

The study of genotoxicity was carried out with *Allium cepa*. Both raw and treated textile effluent was used to treat the roots. The growths after 48 h of incubation at room temperature were examined^32^. A light microscope (NikonH600L Eclipses LV100) was used to obtain mean values of root length, mitotic index (MI) and chromosomal aberrations in the cell. The experiment was conducted in triplicates and the mean ± standard deviation values were accounted.

### Statistical analysis

The toxicity test results for *E. coli* were expressed as EC_50_, which represented a 50% inhibition of *E. coli* growth caused by a percentage concentration of the textile effluent (v/v). EC_50_ was evaluated via the linear interpolation method^33^.

The Linear Regression method is to fit concentration– inhibition data to a linear regression and then to calculate EC_50_ values by linear interpolation. The Interpolation log method is according to Huber and Koella^34^ using the following formula, Eq. (5) to calculate the EC_50_ value.

5 $$\text{log}\left({EC}_{50}\right)=\text{log}\left({x}_{1}\right)+ \frac{50\text{\%}-{y}_{1}}{{y}_{2}-{y}_{1}}\times [\text{log}({x}_{2})-\text{log}({x}_{1})]$$

Direct Interpolation method is similar to interpolation log method but without logarithmic transformation of concentrations. The equation Eq. (6) for EC_50_ calculation is according to ^35^ as follows.

6 $${EC}_{50}={x}_{1}+\frac{50\%-{y}_{1}}{{y}_{2}-{y}_{1}}\left({x}_{2}-{x}_{1}\right)$$

x_1_, x_2_: two conc. of the textile effluent; y_1_, y_2_: corresponding inhibitions (y_1_ < 50%; y_2_ > 50%). % Inhibition can be calculated from the following formula (7)^36^

7 $$\frac{{A_{{control}} - A_{{sample}} }}{{A_{{control}} }} \times 100$$

After evaluation of EC_50,_ the toxicity of all samples were expressed as toxic units, TU (unitless), as per Eq. (8) ^37^,

8 $$TU_{{50}} = 100/EC_{{50}}$$

The Pearson’s correlation coefficient at the significance level of 0.05 was used to assess the correlation between toxicity and conventional indicators of the raw and treated textile effluent, and the impact of COD on toxicity was analyzed by means of linear regression. The significance level of the regression analysis (p) and R^2^ illustrated the extent of toxicity variance caused by COD.

## Results and discussion

### Conventional indicators of textile effluent

The conventional indicators of raw and treated textile effluent have been listed in Table 1. It was evident that the raw textile effluent was high in COD, BOD, TSS and salinity^38^, which was way much higher than the permitted levels (COD- less than 250 mg L^−1^, BOD- less than 30 mg L^−1^, TSS- less than 100 mg L^−1^ in India). Studies show that common components of dye effluents such as some acid dyes and ionic dyes could easily escape into the environment leading to elevating levels of COD, BOD, TSS, salinity and coloration of water bodies^39^. After treatment, it was observed that *G. candidum* was able to remove COD (98.5%), BOD (56.3%), TSS (73.2%), salinity (64%), dye concentration (87%), color (89%) from the textile effluent in 18 h, which meets the discharge standards. Also the pH of the treated effluent was found to drop and reach neutral point. The high removal efficiency of *G. candidum* improved the quality of textile effluent in terms of COD, BOD and color. A recent study showed the reduction in COD, BOD and color of the textile effluent by 77.5%, 71.0% and 99.2% respectively in 24 h after treatment with *Aspergillus niger*^40^. Yet another study showed that 91%, 88% and 68% reduction was recorded in the color intensity, COD and BOD of the textile wastewater, respectively, after treatment with *Peyronellaea prosopidis*^41^.

**Table 1:** Conventional indicators in raw and treated textile effluent.

Indicators	Raw textile effluent	Treated textile effluent (after 18 h)	Removal (%) (after 18 h)
COD (mg L^−1^)	902.1 ± 0.05	13.20 ± 0.05	**98.5**
BOD (mg L^−1^)	350.3 ± 0.02	153.08 ± 0.02	**56.3**
pH	7.9	7	**-**
TSS (mg L^−1^)	320 ± 0.05	85.5 ± 0.05	**73.2**
Salinity (PSU)*	0.25	0.09	**64**
Color (hazen units)	35,400	3894	**89**
Concentration of sample(ppm)	3100	400	**87**

*PSU- Practical Salinity Units.

### Growth kinetics studies

The specific growth rate of 0.127 h^–1^ and yield coefficient of 2.64 mg of dry weight of biomass mg^−1^ COD were obtained for the biodegradation using *G. candidum*. This indicated that *G. candidum* was able to thrive in the extreme conditions of the effluent, reducing COD efficiently. The literatures reported the specific growth rate of 0.116 h^−1^ and yield coefficient of 1.22 mg of dry weight of biomass/mg COD^31^. Yet another report showed the specific growth rate varying from 0.001 to 0.003 h^−1^ and yield coefficient varying from 0.25 to 0.75 mg of dry weight of biomass mg^−1^ COD^42^.

### Characterization of biodegraded textile effluent

**Figure 1:**
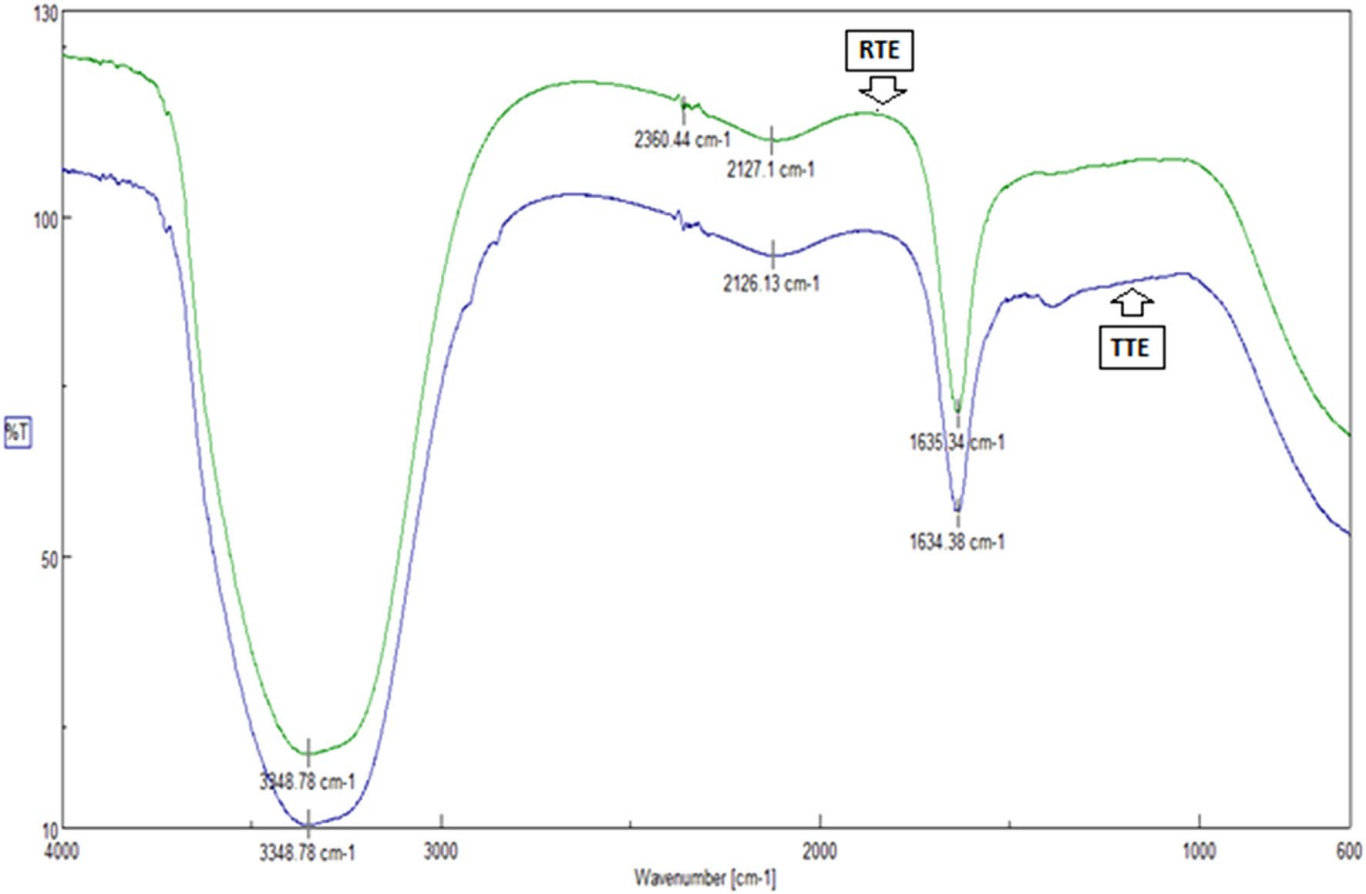
FT-IR spectral analyses of raw and treated textile effluent.

*FTIR* *G. candidum*-induced effluent biodegradation which was established through FTIR spectral analyses (Fig. 1). The process of biodegradation is demonstrated either by loss of absorbance peaks or by the occurrence of new peaks^43,44^. The FTIR spectrum of the untreated effluent represented the variable stretching vibrations of C = C (alkyne), P–H (phosphine) and N–H(amine) at 2127 cm^−1^, 2360 cm^−1^ and 3348 cm^−1^ respectively. The biodegradation products obtained after 18 h of treatment showed disappearance of phosphine group and occurrence of a new peak at 530 cm^−1^, representing the occurrence of strong stretching vibration of C–Br (alkyl bromide) (Fig. 1). The biodegradation of raw textile effluent by *G. candidum* was clearly established in this study. Previous studies have established that the biodegradation of textile dyes can be confirmed by FTIR spectrum representing occurrence of new peaks^44^. The signal in IR at 1634 cm^−1^, which corresponds to aldehyde. Thus aldehyde, one of the intermediate, formed during degradation of acid orange is confirmed. During the degradation there is asymmetric cleavage of azo bond in acid orange resulting in formation of nitrosobenzene, which was confirmed by the standard GC–MS library data, this is further converted to aniline and later on aromatic ring cleavage leading to complete mineralization. While the naphthalene part of the dye was further biodegraded with opening of one ring, the formation of aldehyde as one of the intermediate is confirmed from the IR data. On the basis of above results, it can be concluded that *G. candidum* has ability to mineralize acid orange completely. Similar results were demonstrated by previous studies^45^.

**Figure 2:**
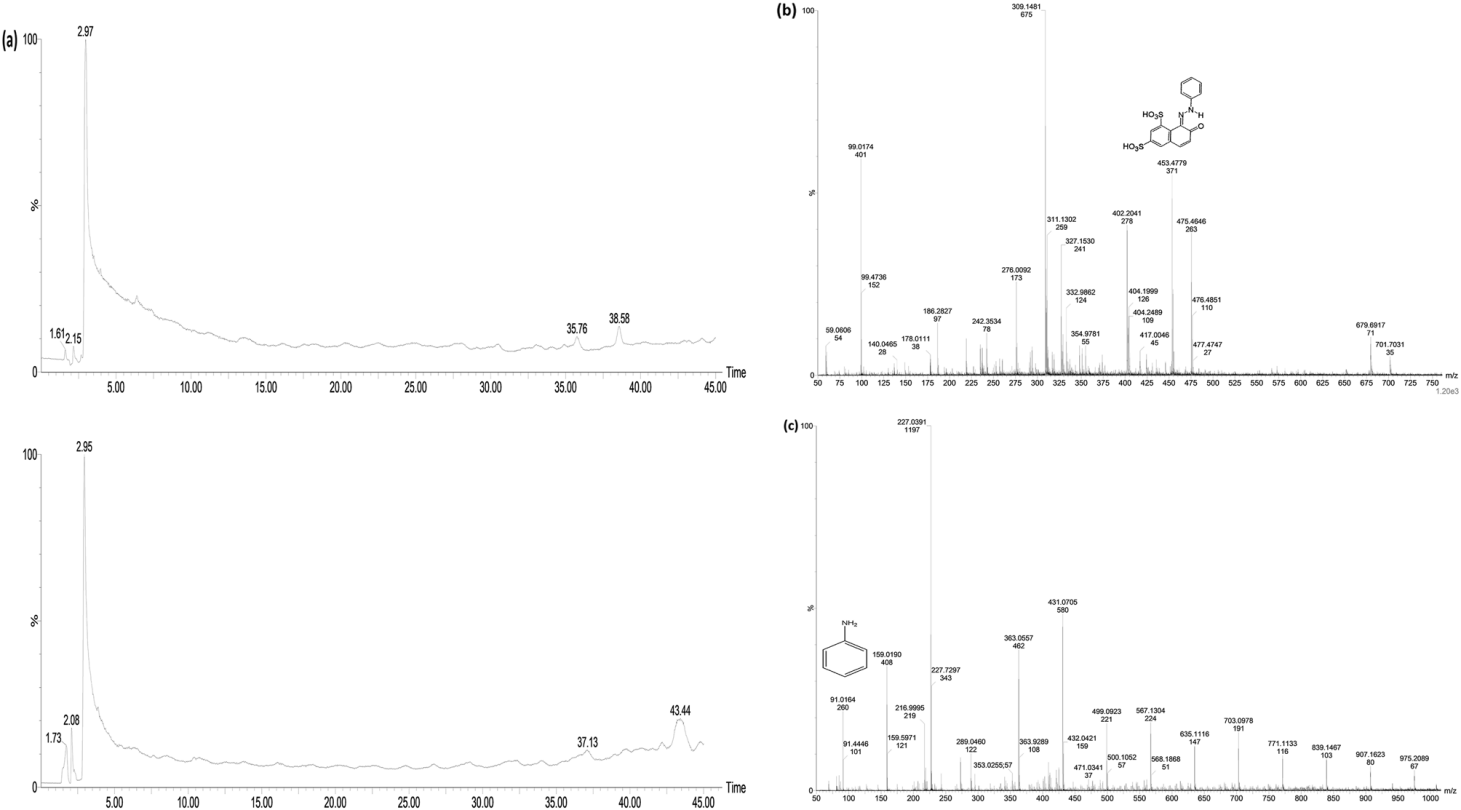
LCMS analysis of raw and treated textile effluent.

**Figure 3:**
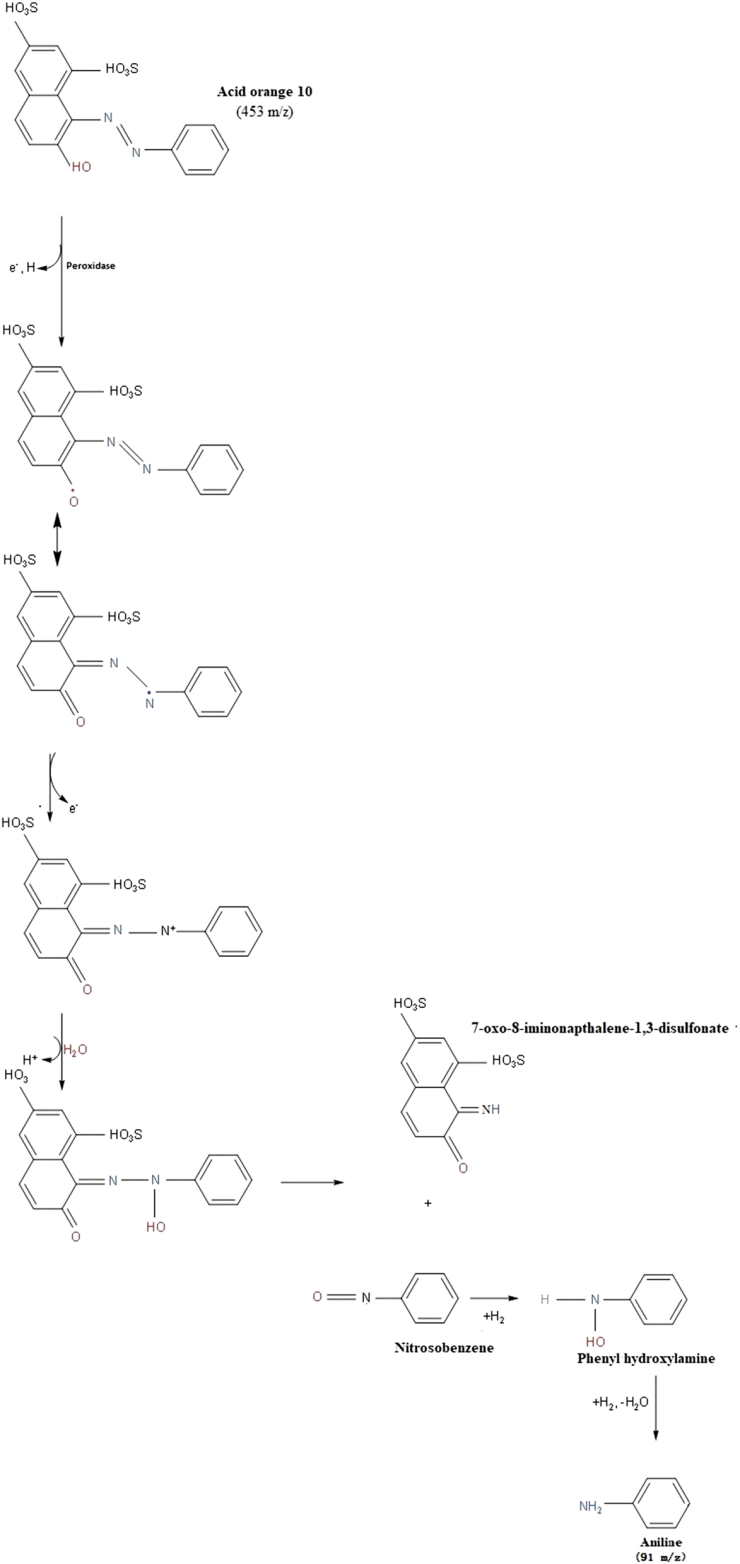
Proposed pathway for degradation of acid orange 10 dye by *G. candidum* (Images have been drawn using the software KingDraw (v1.1.0, http://www.kingdraw.cn/en/download.html).

*LCMS* In positive ion and full scanning mode, the textile dye effluent was analyzed from 100 to 1000 m/z. Several mass peaks of varying values have been observed with both raw and treated effluent (Fig. 2a). For the raw effluent, a mass peak at 453 m/z (m.w. 452) was identical to that of an azo dye, acid orange 10 (Fig. 2b). Hence, it was evident that the acid orange 10 was a major dye component of the textile effluent used in this study. The identification of metabolites produced prior to biodegradation of effluent was carried out and the plausible biodegradation pathway based on the previous reports was predicted^46^. The secretion and involvement of ligninolytic enzymes (laccase and lignin peroxidase) in the azo dye degradation process of *G. candidum* was evident from our previous study^25^. The degradation of acid orange 10 by peroxidase leads to the formation of two biodegradation products such as 7-oxo-8-iminonapthalene -1,3-disulfonate and nitrosobenzene, which subsequently undergoes stepwise hydrogenation and dehydration to form aniline (m.w. 93, m/z 91) (Fig. 2c), via phenyl hydroxyl amine intermediate (as proposed by Mahata and co-workers^47^. The degradation pathway has been predicted in Fig. 3. Aniline was found in the treated effluent as a low molecular weight compound, rendering it less toxic.

**Figure 4:**
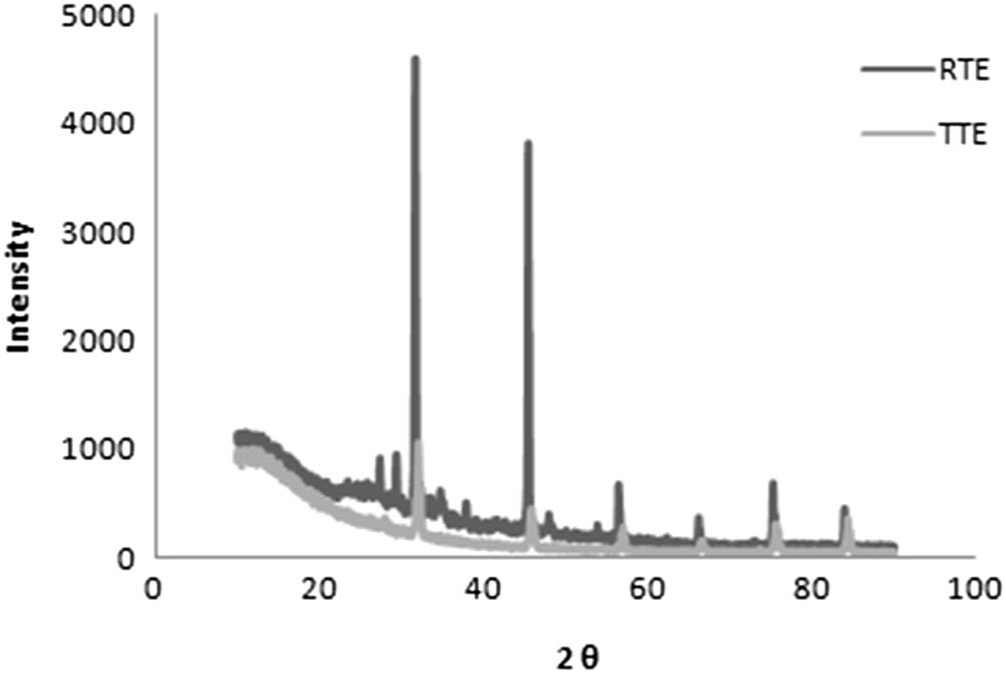
X-ray diffraction pattern of raw and treated textile effluent.

*XRD* Heavy metal ions can be prevalent in textile effluent because of the metal-associated dyes and/or additional components used in the dyeing process. The X-ray spectra obtained for the dried samples of the untreated and treated effluent after 18 h treatment with *G. candidum* are shown in Fig. 4. This displayed various peaks that specifically indicated the presence of metals in raw effluent. The 2θ values of 32.36° and 50.5° show the presence of major metals, such as lead and mercury respectively. The presence of these metals was confirmed using standard JCPDS reference codes (04–0686(Pb) and 01–085-0211(Hg)). Subsequently, the X-ray spectra of treated effluent showed the absence of Pb as well as the considerable decrease in the peak intensity for Hg indicating the decreased toxicity of the effluent (Fig. 4). A decrease in % crystallinity was observed in the textile effluent after it was exposed to *G. candidum*, indicating the degradation of the effluent. Previous studies have demonstrated similar removal of heavy metals from textile effluent^31^. The presence of heavy metals in the effluent leads to numerous health hazards and sequential treatment would eliminate the heavy metals, and make the treated effluent more safe to discharge into the environment.

### Toxicity evaluation

#### Phytotoxicity

**Figure 5:**
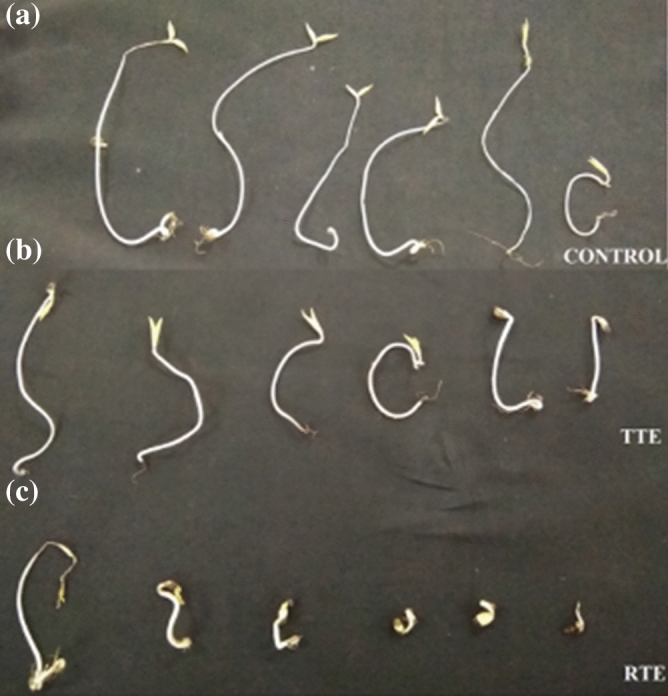
Phytotoxicity analysis of (**a**) Control (Tap water), (**b**) Treated textile effluent, (**c**) Raw textile effluent on *P. mungo*.

Agriculturally valuable seeds i.e. *P. mungo* were used for phytotoxicity assessment of raw and treated textile effluent. The analyzed parameters were germination percentage, plumule and radical length. In control set, i.e., seeds germinated in tap water, 100% germination was observed after 7 days, which reduced to about 33% in case of raw textile effluent (*P* > 0.05) (Fig. 5). Though some of the seeds exposed to raw textile effluent germinated, they couldn’t grow further, exhibiting maximum phytotoxicity. Similar studies on the toxic effects of azo dye-laden textile effluent on the seed germination has been reported^48,49^. The study on inhibitive effect of azo dyes on plants by Zhou and Xiang showed that the azo dyes inhibit the ATPase activity of plants, photosynthetic oxygen evolution and plant growth. In the current study, due to the higher degradation of textile effluent by *G. candidum*, a germination rate of 100% was recorded in *P. mungo* with treated textile effluent. It was indicative that the metabolites formed after effluent biodegradation are less harmful than the compound present in the raw textile effluent. The results shown in Table 2 indicated that the germination (%) and length of plumule and radicle of *P. mungo* seeds were less with the untreated as compared to treated effluent. This study showed that the seed germination and average plumule and radical development were unaffected by decolored textile effluent. A significant decrease (*P* > 0.05) in the average plumule and radical of the germinated seeds was found in the untreated textile effluent. Thus, the comprehensive results indicated that the textile effluent treated with *G. candidum* was not harmful to plant germination and growth. This ensures that treated effluent could be used for agriculture or recycled.

**Table 2:** Phytotoxicity study of untreated and treated textile effluent on *P. mungo*.

Parameters	Control	Raw textile effluent	Treated textile effluent
Germination (%)	98 ± 0.08	33 ± 0.06	95 ± 0.03
Plumule (cm) Mean ± SD	6.86 ± 0.954	0.28 ± 0.577	0.6 ± 0.441
Radicle (cm) Mean ± SD	8.38 ± 2.399	3.15 ± 1.755	6.46 ± 1.503

Values are mean of three experiments, SD ( ±), significantly different from the control (seeds germinated in water) at *P* > 0.05 (One-way analysis of variance, ANOVA).

#### Toxicity test with E. coli

**Figure 6:**
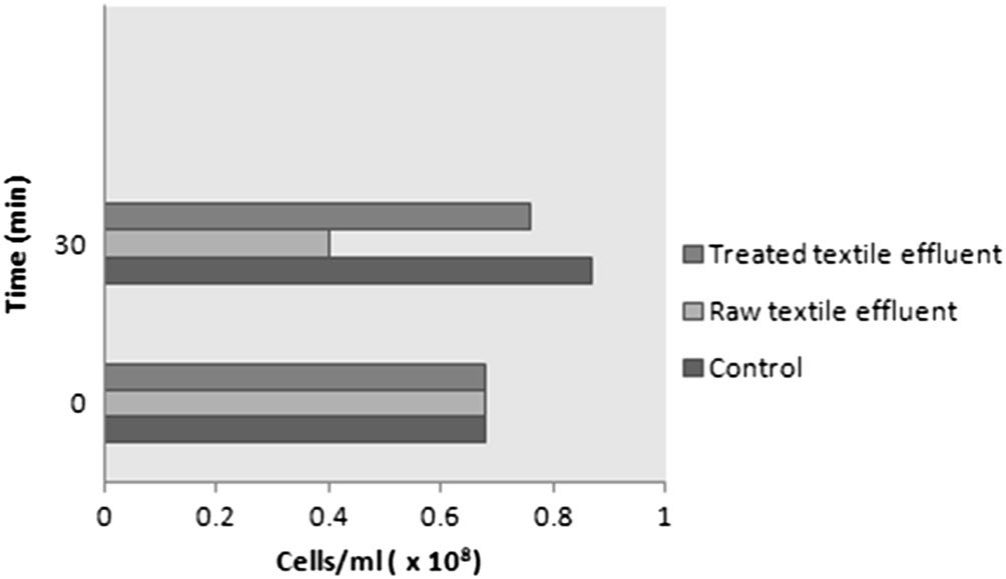
Microbial toxicity analysis of (**a**) Control (culture medium), (**b**) Treated textile effluent, (**c**) Raw textile effluent on *E. coli*.

The short-term toxicity of the raw and treated textile effluent was assessed using the well-established bacteria *E. coli* and the results are shown in Fig. 6. The raw textile effluent was highly toxic, while treated textile effluent presented a low toxicity. Figure 6 shows that after 30 min of exposure to raw textile effluent, the number of bacterial cells declined drastically by approximately 41%, indicating acute toxicity to *E coli.* This was plausibly due to the enormous amount of ionic and acid dyes entering the wastewater during the textile processing. Ionic and disperse dyes discharged from textile processing and dyeing were usually particularly toxic, and some were mutagenic and carcinogenic^50,51^. On the contrary, it was found that the treated effluent was absolutely harmless to the bacteria, which was evident from their uninhibited growth (cells/ml increased by approximately 10%). This indicates that the toxicity of textile effluent was reduced to a greater extent after treatment with *G. candidum*.

#### Genotoxicity analysis

The *A. cepa* study is a standard test to assess the genotoxicity of any toxic substance. The test was carried out to identify MI and chromosomal aberrations in the root cells (Table 3). On the basis of the MI value, (MI value can act as a biosensor for environmental contaminants) the cytotoxic effect of the toxic compound was assessed^52^. Table 3 shows the genotoxic aspects of the textile effluent before and after treatment. The decreased MI value is indicative of decreased cytotoxicity of treated effluent. The textile wastewater typically has a detrimental impact on chromosomal cell division, and this type of aberrations in mitotic cell division is triggered by spindle apparatus proteins malfunctioning^32,53,54^ or probably due to decrease in ATP synthesis during cell division. The significant reduction in COD level could then contribute to the decline in the number of aberrant mitotic cells after treatment. The findings recorded were similar to the literature data^52,54^.

**Table 3:** Genotoxicity analysis for the raw and treated effluent.

Analysis	Raw Effluent	Treated effluent
RL (cm)	3.28 ± 0.65	5.84 ± 0.41
MI	0.3 ± 1.32	0.9 ± 0.562
MN	Not found	Not found
CB	3	1
TA	3	1
TCA	50	50
Frequency of TA	0.5 ± 0.04*	0.25 ± 0.005

RL- root length; MI- Mitotic index; MN- micronuclei; CB- Chromosome breaks; TCA- total no. of cells analysed; TA-Total no. of alterations. Values are mean of three experiments, * P < 0.05, ** P < 0.001 by one-way analysis of variance (ANOVA) with Tukey–Kramer comparison test.

The decrease in colour, COD and BOD might have led to the minimization in the toxicity of textile effluent. This study indicates that the metabolites produced after biodegradation are less toxic than the compounds present in raw effluent.

#### Relationship between toxicity and COD of raw and treated textile effluent

Pearson’s correlation analysis for the textile effluent indicated a significantly positive correlation between COD and TU_50_ (r = 0.920, *P* < 0.05, R^2^ = 0.84), which suggested that compounds present in the textile effluent were toxic to *E. coli*. However, there was a negative relationship between COD and TU_50_ (r = 0.088, *P* = 0.048, R^2^ = 0.77) in case of treated textile effluent. Similar toxicity and COD correlation studies for textile effluent have been carried out with *Vibrio fischeri* and *Desmodesmus subspicatus*. They showed that there was a significant positive correlation between *V. fischeri* and COD; color and TU_50_ (r = 0.824, 0.57, *P* < 0.05), which suggested that compounds producing color might be toxic for *V. fischeri*. However, there was a negative relationship between *D. subspicatus* and TU_50_ (r = 0.625, *P* = 0.035)^55^.

COD is one of the most widely used water quality monitoring metrics and also an important measure for the regulation of the usage of wastewater treatment facilities, taxation and surveillance of wastewater effluent^56^.Therefore, it was important to establish associations between bio-toxicity and conventional markers such as COD^57^.

## Conclusion

The competent *Geotrichum candidum* culture involved in the current work biodegraded the toxic textile effluent. The analysis of conventional parameters such as COD, BOD and color were indicative of the decreased toxicity of the treated effluent in comparison to the raw effluent. The effective decolorization and biodegradation of effluent in 18 h was confirmed by FTIR and XRD analysis. The plausible biodegradation pathway has been proposed on the basis of metabolites identified by LCMS. This demonstrated the first report on the proposed pathway for enzymatic degradation study of acid orange 10 by *G. candidum*. The genotoxicity, phytotoxicity and microbial toxicity analyses proved the raw effluent is harmful, whereas the treated effluent is less toxic. Relationship between effluent COD and TU_50_ showed that an increase in effluent COD resulted in increase in wastewater toxicity. There was a clearly defined correlation between toxicity and COD. It was evident that toxic effects of the textile effluent were significantly reduced upon treatment with *G. candidum.* The major relationships between toxicity and COD will provide directions for more efficient control of textile dyeing effluents. This is the first report on the positive correlation between toxicity and COD of textile effluent using *G. candidum* within a very short stretch of time (18 h). Therefore, the findings of this study have shown that the treated effluent was safer to be released in regard to physicochemical parameters and toxicity unit (TU_50_). The correlation between conventional indicators and toxicity may provide assistance in effluent management.

## Ethics approval

Not applicable.

## Consent to participate

Not applicable.

## Consent for publication

The authors have their consent for the publication of this manuscript if accepted.

## Data Availability

Not applicable.
